# *Desulfovibrio desulfuricans* AY5 Isolated from a Patient with Autism Spectrum Disorder Binds Iron in Low-Soluble Greigite and Pyrite

**DOI:** 10.3390/microorganisms9122558

**Published:** 2021-12-10

**Authors:** Olga V. Karnachuk, Olga P. Ikkert, Marat R. Avakyan, Yurii V. Knyazev, Mikhail N.Volochaev, Viacheslav S. Zyusman, Vasily L. Panov, Vitaly V. Kadnikov, Andrey V. Mardanov, Nikolai V. Ravin

**Affiliations:** 1 Laboratory of Biochemistry and Molecular Biology, Tomsk State University, 634050 Tomsk, Russia; but310@mail.ru (O.P.I.); marat@green.tsu.ru (M.R.A.); sapfiet1@gmail.com (V.S.Z.); kia.98@mail.ru (V.L.P.); 2 Kirensky Institute of Physics, 660036 Krasnoyarsk, Russia; yuvknyazev@mail.ru (Y.V.K.); volochaev91@mail.ru (M.N.); 3 Institute of Bioengineering, Research Center of Biotechnology of the Russian Academy of Sciences, 119071 Moscow, Russia; vkadnikov@bk.ru (V.V.K.); mardanov@biengi.ac.ru (A.V.M.); nravin@biengi.ac.ru (N.V.R.)

**Keywords:** autism spectrum disorders, biomineralisation, *Desulfovibrio desulfuricans*, pyrite, greigite, nitrogenase, urease

## Abstract

The sulphate-reducing bacteria (SRB) of genus *Desulfovibrio* are a group of prokaryotes associated with autism spectrum disorders (ASD). The connection between the elevated numbers of *Desulfovibrio* in the gut of children with ASD compared with healthy children remains unresolved. A conceivable consequence of SRB overgrowth in the gut is the conversion of bioavailable iron into low-soluble crystalline iron sulphides, causing iron deficiency in the organism. In this study, we report the draft genome sequence and physiological features of the first cultivable isolate from a patient with ASD, *Desulfovibrio desulfuricans* strain AY5.The capability of the strain to produce crystalline iron sulphides was studied under different pH conditions. The most notable greigite(Fe_3_S_4_) and pyrite (FeS_2_) formation was revealed at pH 6.0, which suggests that the iron loss due to insoluble sulphide formation may occur in the proximal part of the gastrointestinal tract. Strain AY5 was adapted to grow under nitrogen-limiting conditions by N_2_ fixation. The urease found in the strain’s genome may play a role in resistance to acidic pH.

## 1. Introduction

Autism spectrum disorders (ASD) are a group of neurodevelopmental disorders characterised by deficits in social communications and reciprocal interactions, as well as restrictive and repetitive behaviour. The prevalence of ASD has been increasing significantly, with a current estimate of around 1% of the worldwide population and affecting more male than female individuals [1]. The Global Burden of Disease study revealed 62.2 million people with ASD around the world in 2016 [2]. Recent reports have provided evidence of a link between the gut microbiota and autism-like behaviours [3,4,5]. Microbial transfer therapy has been reported to result in significant improvements of autism-related symptoms [6].

The sulphate-reducing bacteria (SRB) of the genus *Desulfovibrio* have been found to be more abundant in children with ASD compared with healthy children [7,8,9,10]. A strong correlation between *Desulfovibrio* number and the severity of autism manifestation has been reported [11]. *Desulfovibrio* along with *Clostridiales* and *Bacteroidetes* are defined as ASD-associated bacteria [12]. However, plausible mechanisms underlying the connection between the elevated number of SRB and autism remain elusive. An overlooked consequence of *Desulfovibrio* development in the human gut may be its influence on iron metabolism. A relationship between ASD and iron levels/iron deficiency has been reported [13,14]. Sulphide produced by SRB binds iron in the form of low-soluble sulphides, thus reducing its bioavailability. For instance, the solubility of pyrite (FeS_2_) in water at ambient temperature is not measurable [15].

SRB, including *Desulfovibrio*, have an intrinsic capability to bind metals in sulphides due to the formation of a large quantity of hydrogen sulphide in the course of their energy metabolism. The formation of crystalline iron sulphides—pyrite, marcasite, greigite, and mackinawite—in chemically defined media by *Desulfovibrio desulfuricans* was first reported by Rickard in 1969 [16]. Hexagonal pyrrhotite was discovered after long-term (3-month) exposure of hematite surface to *D. desulfuricans* Essex 6 [17]. The chemical form of iron sulphides formed by SRB can be affected by various pH.In the gastrointestinal (GI) tract, the pH ranges from as low as 5.2 to as high as 7.9, depending on the region, and is influenced by diet, transit time, health state, the established microbiome, and the intake of drugs [18,19,20].

The elucidation of the specific mechanism by which SRB are connected with autism has been hampered by the absence of cultivated forms. We have isolated the first SRB, designated strain AY5, from the faeces of a patient with ASD [21]. On the basis of its 16S ribosomal RNA (rRNA) sequence, the strain fell into the species *D. desulfuricans*, however, the genome sequence and physiological traits of strain AY5 remained unstudied. The preliminary experiments in batch culture without pH control showed that strain AY5 could bind iron in the form of crystalline sulphides. In this study, we investigated the impact of a range of pH conditions on iron sulphide production by strain AY5 in a pH-stat bioreactor and batch culture. The draft genome sequence of strain AY5 has been obtained to check its taxonomic position and physiological features.

## 2. Materials and Methods

### 2.1. Strain AY5 Cultivation and Physiological Tests

Strain AY5 was isolated from a faecal sample of a child (11 years old) with ASD as described previously [21]. Strain AY5 was cultivated in liquid freshwater Widdel and Bak (WB) medium [22], that contained (per litre) 4.0 g Na_2_SO_4_, 0.2 g KH_2_PO_4_, 0.25 g NH_4_Cl, 1 g NaCl, 0.4 g MgCl_2_·6H_2_O, 0.5 g KCl, 0.113 g CaCl_2_, 2 mL of vitamin solution, 1 mL of trace element solution, and 1 mL each of Na_2_SeO_3_ (final concentration of 23.6 µM) and Na_2_WO_4_(final concentration of 24.2 µM) solutions. Vitamin, trace element, and Na_2_S·9H_2_O solutions were prepared and applied as described by *Widdel* and *Bak* [22]. Each cultivation vial received an iron wire (100% Fe) as described previously [23,24]. Glycerol (11 mM) was used as an electron donor. Growth was analysed with the following electron donors: 7.5 mM formate; 7 mM pyruvate; 4.5 mM succinate; 9 mM fumarate; 7.5 mM malate; 5 mM fructose; 5 mM glucose; 3 mM sucrose; 25 mM ethanol; 17 mM propanol; 13.5 mM butanol. Carbohydrate stock solutions were sterilised by using polyethersulfone 0.22 µm Millex-GP filter units (Merck Millipore, Darmstadt, Germany). If growth was observed, the culture was subcultured at least five times in the presence of each electron donor and acceptor to confirm their utilisation.

Cell morphology was observed with phase-contrast microscopy using an Axio Imager A1 microscope and with transmission electron microscopy (TEM) of ultrathin sections prepared as described previously [25]. The TEM and microdiffraction investigations were carried out on a Hitachi HT7700 transmission electron microscope equipped with a microanalysis system with an energy-dispersive X-ray spectrometer (EDS). Micrographs were obtained at an accelerating voltage of 100 kV. The accumulation time for EDS analysis was determined by the quality of the spectrum assembly, which allows for quantitative processing, and was no less than 10 min.

### 2.2. Strain AY5 Genome Sequencing

Genomic DNA was isolated using the Power Soil DNA Isolation Kit (MO BIO Laboratories, Carlsbad, CA, USA) and sequenced using the Illumina platform. The shotgun genome library was prepared using the NEBNext Ultra II DNA library prep kit (New England BioLabs, Ipswitch, MA, USA). The sequencing of this library on an Illumina MiSeq using MiSeq Reagent Kit v3 (2 × 300 nt mode) sequencing reagents generated 2,472,699 paired-end reads. Overlapping paired-end reads were merged using FLASH v1.2.11 [26], and low-quality bases were trimmed using sickle v1.33 (https://github.com/najoshi/sickle/acessed, accessed on 9 December 2021). Illumina reads were assembled into contigs using SPAdes v.3.11.1 [27]. Gene searching and annotation were performed using the RAST server [28].

### 2.3. Bioreactor Culture and pH Gradient

Experiments on iron sulphide formation at different pH were run in a bioreactor.The glycerol-fed strain AY5 culture was used as the starter culture in a Biostat B plus benchtop bioreactor (Sartorius Stedim Biotech GmbH, Göttingen, Germany) with an initial working volume of 1 L, agitation at 100 rpm, and with pH and temperature control. Ultra-pure (99.9%) N_2_ was sparged at 25 mL min^−1^ through a diffuser at the bottom of the bioreactor to maintain O_2_-free headspace. WB medium supplemented with 100 mgL^−1^ iron as FeSO_4_ was used for bioreactor cultivation. The bioreactor was operated at 28 °C by recirculating water through the reactor mantle. The initial pH of 7.0 was controlled by pH stats with 0.5 M HCl or 1 M NaHCO_3_solutions. Samples from the bioreactor were withdrawn daily to measure H_2_S and the cell number, and to collect the precipitate formed in the bioreactor for X-ray diffraction (XRD) analysis.

**Figure 1:**
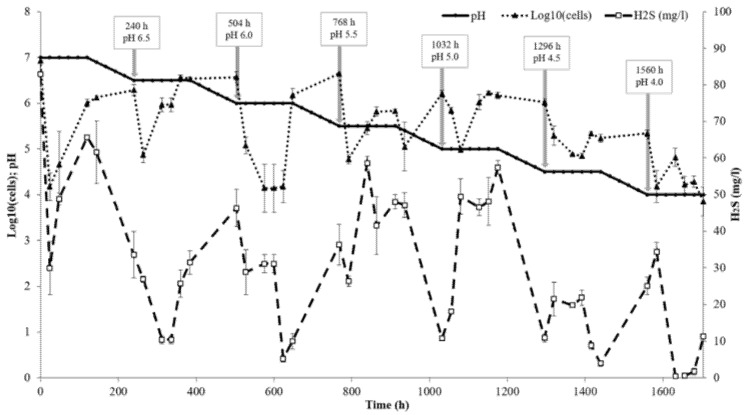
Bioreactor performance: the aliquot withdrawal points and the pH at each point are shown by arrows. The cell number and H_2_S concentration are expressed as the means of three replicates and the vertical bars show the standard deviation.

After 5 days, 0.5 L aliquots were withdrawn from the reactor and distributed into serum bottles tightly closed with rubber lids without a gas phase. The bottles were kept in the dark at 28 °C as batch cultures for a long-time exposure of 8, 18, 24, and 32 days. The bioreactor was operated in the semibatch culture mode: 0.5 L aliquots of fresh glycerol medium were added after aliquot withdrawal and the pH in the bioreactor culture was decreased by 0.1 unit per day by pH stats with 0.5 M HCl or 1 M NaHCO_3_solutions. The same scheme was applied to reduce the pH gradually from 7.0 to 4.0 with concomitant mineralogical analysis of the solid phase. Cultivation in the bioreactor was stopped when strainAY5 cells were lysed at pH 3.7 (Figure 1).

### 2.4. XRD

Precipitates from the experiment were harvested by centrifugation (13,100× *g*, 15 min, 15 °C). The precipitate was washed with distilled water (10 min) to remove any loosely associated and unsequestered metals. Powder XRD was performed with a Rigaku Ultima 4 diffractometer (Rigaku Corp., Tokyo, Japan) with CuKα radiation. The samples were packed into zero-background quartz sample holders and step-scanned at the 20 range from 10° to 75° using a 20-step interval of 0.02° and a counting time of 0.8 s. The diffraction patterns were analysed with the Crystallographica-Search Match software and the PDF-4 database (International Centre for Diffraction Data, http://www.icdd.com/acessed, accessed on 9 December 2021).

## 3. Results

### 3.1. Strain AY5 Genome and Physiological Properties

**Figure 2:**
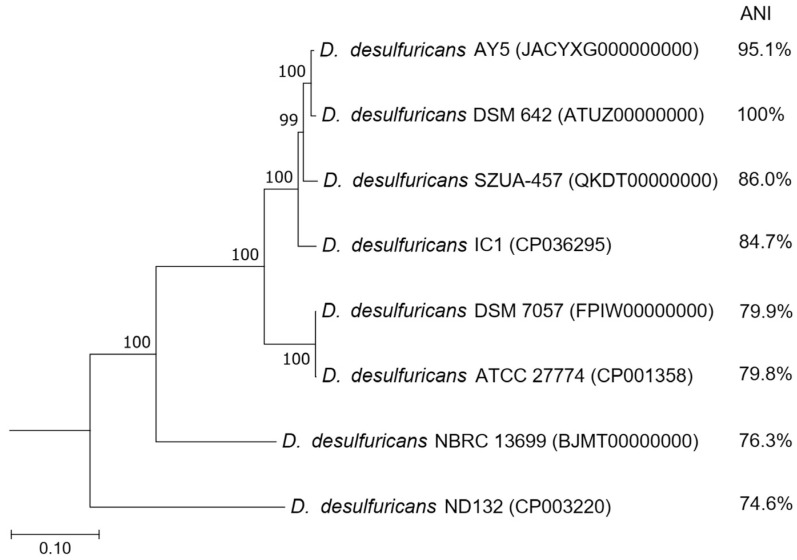
Neighbour-joining tree of concatenated sequences of 120 bacterial single-copy marker proteins. The optimal tree with the sum of the branch length of 1.26840156 is shown. The percentage of replicate trees in which the associated taxa cluster together in the bootstrap test (1000 replicates) is shown next to the branches. The tree is drawn to scale, with branch lengths in the same units as those of the evolutionary distances used to infer the phylogenetic tree. The evolutionary distances were computed by using the Poisson correction method and are presented as the number of amino acid substitutions per site. All positions containing gaps and missing data were eliminated (complete deletion option). There are a total of 3317 positions in the final dataset. *Desulfotomaculum acetoxidans* DSM 771 serves as an outgroup (not shown). Evolutionary analyses were conducted in MEGA X.

The draft genome sequence of strain AY5 consists of 40 contigs with a total size of 3,519,154bp, a G+C content of 57.2%, and an N_50_contig size of 373,881bp. CheckM [29] estimated completeness of the draft genome as 99.41%. The genome contains 3.394coding sequences (CDSs), 9 rRNAs, and 52 transfer RNAs (tRNAs). In the previous study, the taxonomic position of strain AY5 was revealed based on the 16S rRNA gene only, which assumed that the strain belongs to *D. desulfuricans*, with 99.7% sequence similarity between strain AY5 and strain DSM642^T^ [21]. The draft genome sequence allowed us to verify the strain taxonomy. Taxonomic assignment of strain AY5 via a search against GTDB [30] placed it in *D. desulfuricans,* with the most similarity to strain DSM642^T^ (GenBank accession number GCA_000420465.1), the type strain of the species. The average nucleotide identity (ANI) with the *D. desulfuricans* type strain is 95.1%, a value close to the 95% cutoff most frequently used for species demarcation [31]. Thus, the genome analysis confirmed the taxonomic position of strain AY5 within the species *D. desulfuricans* (Figure 2).

A notable feature of strain AY5 is the presence of two gene clusters encoding molybdenum–iron and iron–iron nitrogenases. The two loci are located on the chromosome but distantly from each other. These two types of nitrogenases are also encoded in the genomes of two *Desulfovibrio* species close to strain AY5, namely *Desulfovibrio legallii* and *Desulfovibrio intestinalis*. The amino acid sequence identities of the corresponding NifD alpha subunits between AY5 and the other two species are above 89%. The search against GenBank revealed NifD proteins from *Clostridia* as the next hits, while NifD from other *Desulfovibrio* are distant. Overall, these observations indicate that both *nif* operons were laterally acquired from *Firmicutes* by a common ancestor of *D. desulfuricans*, *D. legallii*, and *D. intestinalis*.

Urea could probably serve as another source of nitrogen for strain AY5, as evidenced by the presence of genetic determinants of urea utilisation, including the urease operon, enabling the hydrolysis of urea into carbon dioxide and ammonia. The operon includes the *ureABC* genes encoding the urease enzyme and the genes *ureEFGD* encoding auxiliary proteins [32]. The urease operon is clustered with the *urtABCDE* operon, which encodes the urea ABC transporter induced under nitrogen deficiency [33]. The search against GenBank revealed that highly similar genes (>90% identity of amino acid sequences of deduced UreA and UrtA) are also present in the genome of *D. legallii* but not in other *Desulfovibrio* species. The next closest hits were with various *Gammaproteobacteria*, suggesting that the urease cluster was acquired via lateral gene transfer.

**Figure 3:**
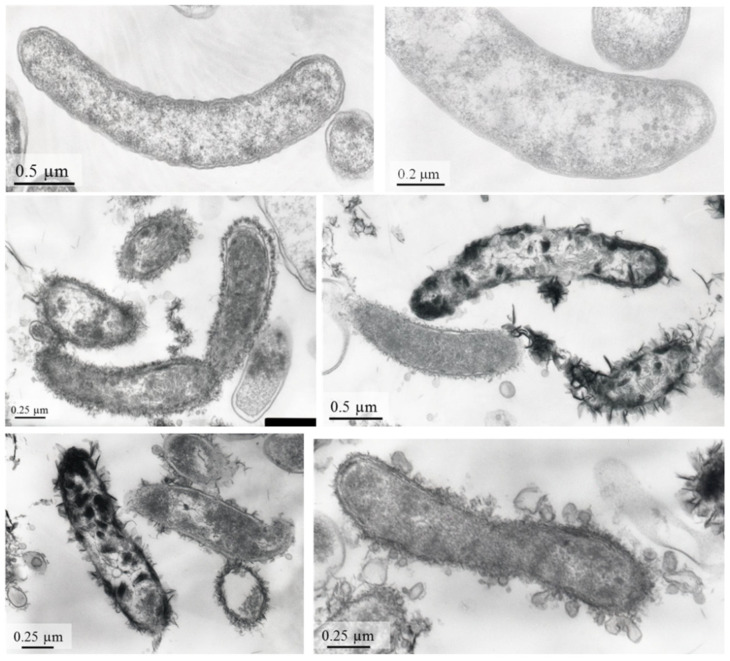
TEM micrographs of ultrathin layers of strain AY5.

Physiological experiments showed that strain AY5 could use lactate, pyruvate, fumarate, ethanol, glycerol, and choline as electron donors for sulphate reduction. Limited growth of the *strain* was observed with succinate, fructose, glucose, and sucrose. Cells of strain AY5 appeared as motile vibrios, 2.2–3.0 μm long and 0.4–0.7 μm wide (Figure 3).

**Figure 4:**
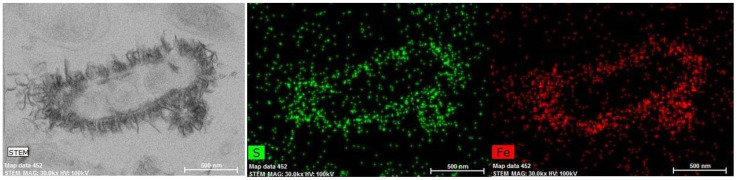
Transmission electron micrographs and energy-dispersive X-ray spectrometry mapping of ultrathin cross-sections of *Desulfovibrio desulfuricans* strain AY5 cell showing electron-dense flake-like particles adhering to the cell wall and electron maps of Fe and S in the same image site with the same magnification.

TEM of ultrathin sections revealed numerous outer membrane vesicles in the cells from the late stationary phase. Electron micrographs also showed electron-dense flake-like particles adhering to the cell walls and often appeared as clusters. Energy-dispersive analysis and elemental mapping showed that the electron-dense particles were highly enriched in S and Fe (Figure 4). The presumed iron sulphide precipitate had already formed in the exponential growth phase on the second day of strain AY5 cultivation in the presence of 100 mgL^−1^ Fe (Figure S1).

### 3.2. Low-Soluble Sulphide Production by Strain AY5 at Different pH

**Figure 5:**
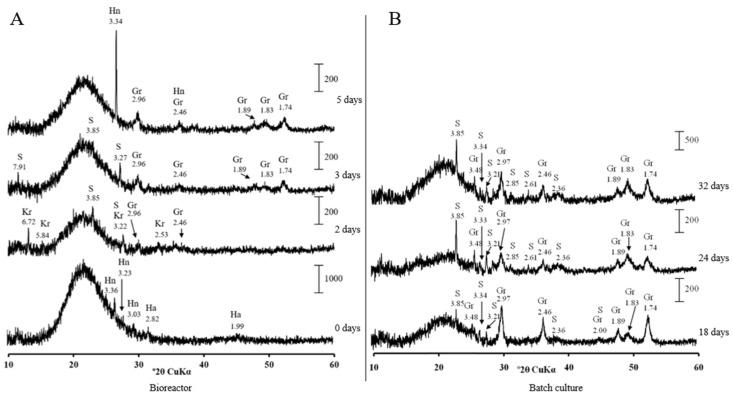
X-ray diffraction patterns of solids from the bioreactor (**A**) and batch cultures (**B**) of *Desulfovibrio desulfuricans* strain AY5 grown at pH 7.0 after different incubation times. Letter codes: Gr = greigite, Fe_3_S_4_(PDF-16-0713); S = sulphur (PDF-06-0248); Hn = hentschelite, CuFe_2_(PO_4_)_2_(OH)_2_(PDF-78-1104); Kr = kornelite, Fe_2_^+3^(SO_4_)_3_·7H_2_O (PDF-44-1426).

Mineralogical analysis of precipitates from the bioreactor at pH 7.0 showed no crystalline phases until day 2, when tiny peaks characteristic of greigite (Fe_3_S_4_) appeared (Figure 5A). Greigite increased in crystallinity by day 5 in the bioreactor. Several well-resolved peaks of greigite were observed after 8 days of cultivation in the batch-culture mode (Figure 5B).

No other crystalline sulphides were formed by strain AY5 at pH 7.0. No pronounced crystalline phase was observed in precipitates from the bioreactor when strain AY5 was grown at pH 6.5 (Figure S2A). The greigite peaks appeared in the precipitates when the culture was transferred to the batch culture mode by day 8 (Figure S2B). An iron phosphate, vivianite, Fe_3_(PO_4_)_2_·8H_2_O, was the major crystalline phase starting from day 24 in the batch culture.

**Figure 6:**
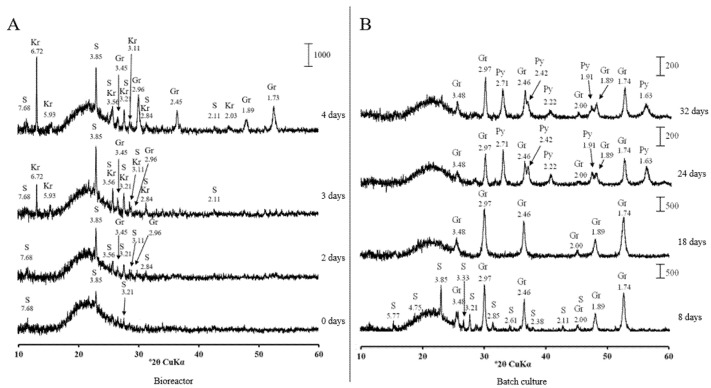
X-ray diffraction patterns of solids from the bioreactor (**A**) and batch cultures (**B**) of *Desulfovibrio desulfuricans* strain AY5 grown at pH 6.0 after different incubation times. Letter codes: Gr = greigite, Fe_3_S_4_(PDF-16-0713); Py = pyrite, FeS_2_(PDF-42-1340); S = sulphur (PDF-06-0248); Kr = kornelite, Fe_2_^+3^(SO_4_)_3_·7H_2_O (PDF-44-1426).

The most pronounced crystalline sulphide formation was detected when the pH in bioreactor was decreased to 6.0. The well-resolved peaks of greigite were observed by days 2 and 3 (Figure 6A). Greigite gained in crystallinity by days 8 and 18, when the culture was transferred to the batch conditions (Figure 6B).

**Figure 7:**
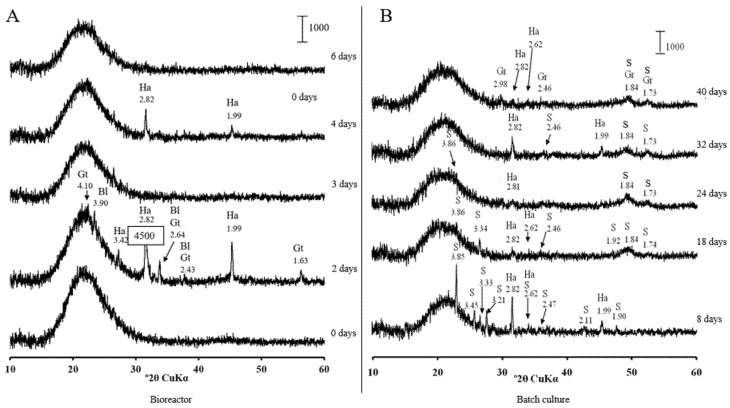
X-ray diffraction patterns of solids from the bioreactor (**A**) and batch cultures (**B**) of *Desulfovibrio desulfuricans* strain AY5 grown at pH 5.0 after different incubation times. Letter codes: Gr = greigite, Fe_3_S_4_(PDF-16-0713); Ha = halite, NaCl (PDF-05-0628); Bl = bernalite, Fe^3+^(OH)_3_(PDF-46-1436); Gt = goethite, Fe^+3^O(OH)(PDF-03-0251); S = sulphur (PDF-08-0248).

Table S2 appeared by day 24 in batch culture. Greigite was the major crystalline phase in the precipitate at pH 5.5. It appeared by day 1 of cultivation in the bioreactor (Figure S3). Further reduction in pH in the bioreactor to 5.0 resulted in the disappearance of crystalline sulphides. Poorly resolved peaks of greigite appeared by day 40, when the culture was transferred to the batch conditions (Figure 7).

## 4. Discussion

Apart from the type strain, six genomes of *D. desulfuricans* are available in the NCBI database. The phylogenetic analysis of concatenated sequences of 120 conservative marker genes from the genomes revealed that strains DSM 7057 (GCA_900119095.1) and ATCC27774 (GCA_000022125.1) form a separate cluster on the tree, and possibly represent different species (Figure 2). The ANI value between these strains and the type strain, DSM642^T^, does not exceed 80%. The same refers to the strains NBRC 13,699 (GCA_006539305.1) and ND132 (GCA_004801255.1), indicating that their taxonomic position may require reclassification.

The occurrence of the complete nitrogenase operon in the strain AY5 genome and its capability to grow in an N_2_atmosphere without any nitrogen addition to the liquid medium is in line with the suggestion that N_2_fixation by strain AY5 may be beneficial for its growth in the human gut. Animals are often nitrogen-limited, and overexpression of nitrogenase genes was demonstrated in the first human isolate, *Desulfovibrio diazotrophicus*, capable of nitrogen fixation [34]. The transcriptome analysis of *D. diazotrophicus* grown in nitrogen-limiting conditions revealed overexpression of a urea uptake system and the urease complex along with the nitrogenase complex. Urease transforms urea into ammonia and CO_2_, a phenomenon that also increases the pH due to the alkaline properties of ammonia. *Desulfovibrio* most likely acquired the urease complex from *Gammaproteobacteria* by horizontal gene transfer. The ability of ureases to raise the pH of their environment benefits pathogens, including the notorious *Helicobacter pylori*, when colonising the stomach and downstream gut [35]. It is conceivable that strain AY5 may utilise ammonia production via urease activity to survive at a low pH in the upper GI tract.

The significant pH alteration in the different sections of human intestine was demonstrated with the use of a telemetric drug delivery device [19]. In the proximal part, the pH varies from 5.9 to 6.3 and increases to 7.4–7.8 in the distal part. In our experiments in the bioreactor and batch culture, the most pronounced crystalline iron sulphide formation occurred at pH 6.0. This implies that the most significant iron loss due to insoluble sulphide formation occurs in the proximal part of the GI tract. In the stomach, where the pH is less than 5, and in the distal intestine, where the pH exceeds 7, the conditions do not benefit crystalline sulphide production. Fe^2+^ absorption occurs mainly in the proximal duodenum at the brush border of the mucosa cells [36].

It is conceivable that *Desulfovibrio* associated with the mucosa provides initial nucleation sites for the formation of iron sulphide nanocrystals. The observed Fe-S-containing layer surrounding the strain AY5 cells may provide a nanoparticulate phase, which was demonstrated as a necessary solid-phase precursor prior to the formation of stable iron sulphide crystals [37]. The mucus layer, covering the epithelial cells of the gut, contains sulphated oligosaccharides [38]. Sulphate derived from sulphomucin degradation may promote the colonization of the gut by SRB, including *Desulfovibrio* [39]. SRB are a common constituent of the human gut microbiome [40,41]. Relatively little is known about the ecology of SRB populations in the human colon; however, in the mouse, SRB were found to be most abundant in those intestinal regions harbouring the greatest density of sulphomucin-containing goblet cells [42].

A recent report of a large autism stool metagenomics study (*n* =247) questioned direct associations between ASD diagnosis and the gut microbiome [43]. Instead, an ASD-related less-diverse diet was suggested to cause reduced microbial taxonomic diversity. From this point of view, the dietary requirements preventing iron binding in the GI tract by SRB are conceivable. Nonetheless, further investigation into the role of bioavailable iron reduction in the human colon via microbial sulphate reduction is warranted.

## Data Availability

The entire genome shotgun project has been deposited at DDBJ/ENA/GenBank under the BioProject number PRJNA666287 and accession number JACYXG000000000. The version described in this paper is version JACYXG010000000. The raw reads are available under the SRA accession number SRR12825170.

## Supplementary Materials

The following are available online at https://www.mdpi.com/article/10.3390/microorganisms9122558/s1, Figure S1. The presumed iron sulphide precipitate formed already in the exponential growth phase on the second day of *Desulfovibriodesulfuricans* strain AY5 cultivation in the presence of 100 mgL^−1^ Fe. Figure S2. X-ray diffraction patterns of solids from bioreactor and batch cultures of *Desulfovibrio desulfuricans* strain AY5 grown at pH 6.5 after different incubation time. Figure S3. X-ray diffraction patterns of solids from bioreactor and batch cultures of strain AY5 grown at pH5.5 after different incubation time.

## References

[1] Catalá-López F., Ridao M., Hurtado I., Núñez-Beltrán A., Gènova-Maleras R., Alonso-Arroyo A., Tobías A., Aleixandre-Benavent R., Catalá M.A., Tabarés-Seisdedos R. (2019). Prevalence and Comorbidity of Autism Spectrum Disorder in Spain: Study Protocol for a Systematic Review and Meta-Analysis of Observational Studies. Syst. Rev..

[2] GBD 2016 Disease and Injury Incidence and Prevalence Collaborators (2017). Global, Regional, and National Incidence, Prevalence, and Years Lived with Disability for 328 Diseases and Injuries for 195 Countries, 1990–2016: A Systematic Analysis for the Global Burden of Disease Study 2016. Lancet.

[3] Srikantha P., Mohajeri M.H. (2019). The Possible Role of the Microbiota-Gut-Brain-Axis in Autism Spectrum Disorder. Int. J. Mol. Sci..

[4] Bezawada N., Phang T.H., Hold G.L., Hansen R. (2020). Autism Spectrum Disorder and the Gut Microbiota in Children: A Systematic Review. Ann. Nutr. Metab..

[5] Johnson D., Letchumanan V., Thurairajasingam S., Lee L.H. (2020). A Revolutionizing Approach to Autism Spectrum Disorder using the Microbiome. Nutrients.

[6] Kang D.W., Adams J.B., Coleman D.M., Pollard E.L., Maldonado J., McDonough-Means S., Caporaso J.G., Krajmalnik-Brown R. (2019). Long-Term Benefit of Microbiota Transfer Therapy on Autism Symptoms and Gut Microbiota. Sci. Rep..

[7] Finegold S.M. (2011). *Desulfovibrio* Species Are Potentially Important in Regressive Autism. Med. Hypotheses.

[8] Finegold S.M., Downes J., Summanen P.H. (2012). Microbiology of Regressive Autism. Anaerobe.

[9] De Angelis M., Piccolo M., Vannini L., Siragusa S., De Giacomo A., Serrazzanetti D.I., Cristofori F., Guerzoni M.E., Gobbetti M., Francavilla R. (2013). Fecal Microbiota and Metabolome of Children with Autism and Pervasive Developmental Disorder Not Otherwise Specified. PLoS ONE.

[10] Liu F., Li J., Wu F., Zheng H., Peng Q., Zhou H. (2019). Altered Composition and Function of Intestinal Microbiota in Autism Spectrum Disorders: A Systematic Review. Transl. Psychiatry.

[11] Tomova A., Husarova V., Lakatosova S., Bakos J., Vlkova B., Babinska K., Ostatnikova D. (2015). Gastrointestinal Microbiota in Children with Autism in Slovakia. Physiol. Behav..

[12] Ding H.T., Taur Y., Walkup J.T. (2017). Gut Microbiota and Autism: Key Concepts and Findings. J. Autism Dev. Disord..

[13] Sidrak S., Yoong T., Woolfenden S. (2014). Iron Deficiency in Children with Global Developmental Delay and Autism Spectrum Disorder. J. Paediatr. Child Health.

[14] Yanagimoto Y., Ishizaki Y., Kaneko K. (2020). Iron Deficiency Anemia, Stunted Growth, and Developmental Delay Due to Avoidant/Restrictive Food Intake Disorder by Restricted Eating in Autism Spectrum Disorder. Biopsychosoc. Med..

[15] Rickard D., Morse J.W. (2005). Acid Volatile Sulfide (AVS). Mar. Chem..

[16] Rickard D. (1969). The Microbiological Formation of Iron Sulphides. Stockh. Contrib. Geology.

[17] Neal A.L., Techkarnjanaruk S., Dohnalkova A., McCready D., Peyton B.M., Geesey G.G. (2001). Iron Sulfides and Sulfur Species Produced at Hematite Surfaces in the Presence of Sulfate-Reducing Bacteria. Geochim. Cosmochim. Acta.

[18] Smith E.A., Macfarlane G.T. (1998). Enumeration of Amino Acid Fermenting Bacteria in the Human Large Intestine: Effects of pH and Starch on Peptide Metabolism and Dissimilation of Amino Acids. FEMS Microbiol. Ecol..

[19] Koziolek M., Grimm M., Becker D., Iordanov V., Zou H., Shimizu J., Wanke C., Garbacz G., Weitschies W. (2015). Investigation of pH and Temperature Profiles in the GI Tract of Fasted Human Subjects Using the Intellicap^®^ System. J. Pharm. Sci..

[20] Wetzel D., McBride S.M. (2020). The Impact of pH on Clostridioides Difficile Sporulation and Physiology. Appl. Environ. Microbiol..

[21] Ikkert O.P., Ivanov M.V., Ukhova A., Zuysman V.S., Glukhova L.B., Avakyan M.R., Karnachuk O.V. (2021). *Desulfovibrio* Isolate from the Microbiote of Children with Autistic Spectrum Disorders Immobilizes Iron in Poorly Soluble Crystalline Sulfides. Microbiology.

[22] Widdel F., Bak F., Balows A. (1992). Gram Negative Mesophilic Sulfate Reducing Bacteria. The Prokaryotes: A Handbook on the Biology of Bacteria: Ecophysiology, Isolation, Identification, Applications.

[23] Karnachuk O.V., Pimenov N.V., Iusupov S.K., Frank I.A., Puhakka J.A., Ivanov M.V. (2006). Distribution, Diversity, and Activity of Sulfate-Reducing Bacteria in the Water Column in Gek-Gel Lake, Azerbaijan. Mikrobiologiia.

[24] Karnachuk O.V., Frank Y.A., Lukina A.P., Kadnikov V.V., Beletsky A.V., Mardanov A.V., Ravin N.V. (2019). Domestication of Previously Uncultivated *Candidatus* DesulforudisAudaxviator from a Deep Aquifer in Siberia Sheds Light on Its Physiology and Evolution. ISME J..

[25] Ikkert O.P., Gerasimchuk A.L., Bukhtiyarova P.A., Tuovinen O.H., Karnachuk O.V. (2013). Characterization of Precipitates Formed by H_2_S-Producing, Cu-Resistant Firmicute Isolates of *Tissierella* from Human Gut and *Desulfosporosinus* from Mine Waste. Antonie Van Leeuwenhoek.

[26] Magoč T., Salzberg S.L. (2011). FLASH: Fast Length Adjustment of Short Reads to Improve Genome Assemblies. Bioinformatics.

[27] Nurk S., Bankevich A., Antipov D., Gurevich A.A., Korobeynikov A., Lapidus A., Prjibelski A.D., Pyshkin A., Sirotkin A., Sirotkin Y. (2013). Assembling Single-Cell Genomes and Mini-Metagenomes from Chimeric MDA Products. J. Comput. Biol..

[28] Brettin T., Davis J.J., Disz T., Edwards R.A., Gerdes S., Olsen G.J., Olson R., Overbeek R., Parrello B., Pusch G.D. (2015). RASTtk: A Modular and Extensible Implementation of the RAST Algorithm for Building Custom Annotation Pipelines and Annotating Batches of Genomes. Sci. Rep..

[29] Parks D.H., Imelfort M., Skennerton C.T., Hugenholtz P., Tyson G.W. (2015). CheckM: Assessing the Quality of Microbial Genomes Recovered from Isolates, Single Cells, and Metagenomes. Genome Res..

[30] Chaumeil P.A., Mussig A.J., Hugenholtz P., Parks D.H. (2019). GTDB-Tk: A Toolkit to Classify Genomes with the Genome Taxonomy Database. Bioinformatics.

[31] Jain C., Rodriguez-R L.M., Phillippy A.M., Konstantinidis K.T., Aluru S. (2018). High Throughput ANI Analysis of 90K Prokaryotic Genomes Reveals Clear Species Boundaries. Nat. Commun..

[32] Mobley H.L., Island M.D., Hausinger R.P. (1995). Molecular Biology of Microbial Ureases. Microbiol. Rev..

[33] Beckers G., Bendt A.K., Krämer R., Burkovski A. (2004). Molecular Identification of the Urea Uptake System and Transcriptional Analysis of Urea Transporter- and Urease-Encoding Genes in Corynebacterium Glutamicum. J. Bacteriol..

[34] Sayavedra L., Li T., Batista M.B., Seah B.K.B., Booth C., Zhai Q., Chen W., Narbad A. (2021). *Desulfovibriodiazotrophicus* sp. nov., a Sulfate-Reducing Bacterium from the Human Gut Capable of Nitrogen Fixation. Environ. Microbiol..

[35] Righetto R.D., Anton L., Adaixo R., Jakob R.P., Zivanov J., Mahi M.A., Ringler P., Schwede T., Maier T., Stahlberg H. (2020). High-resolution Cryo-EM Structure of Urease from the Pathogen *Yersinia Enterocolitica*. Nat. Commun..

[36] Talarico V., Giancotti L., Mazza G.A., Miniero R., Bertini M. (2021). Iron Deficiency Anemia in Celiac Disease. Nutrients.

[37] Matamoros-Veloza A., Cespedes O., Johnson B.R.G., Stawski T.M., Terranova U., de Leeuw N.H., Benning L.G. (2018). A Highly Reactive Precursor in the Ironsulfide System. Nat. Commun..

[38] Sicard J.F., Le Bihan G., Vogeleer P., Jacques M., Harel J. (2017). Interactions of Intestinal Bacteria with Components of the Intestinal Mucus. Front. Cell Infect. Microbiol..

[39] Croix J.A., Carbonero F., Nava G.M., Russell M., Greenberg E., Gaskins H.R. (2011). On the Relationship between Sialomucin and Sulfomucin Expression and Hydrogenotrophic Microbes in the Human Colonic Mucosa. PLoS ONE.

[40] Barton L.L., Ritz N.L., Fauque G.D., Lin H.C. (2017). Sulfur Cycling and the Intestinal Microbiome. Dig. Dis. Sci..

[41] Kushkevych I., Martínková K., Vítězová M., Rittmann S.K.R. (2021). Intestinal Microbiota and Perspectives of the Use of Meta-Analysis for Comparison of Ulcerative Colitis Studies. J. Clin. Med..

[42] Deplancke B., Hristova K.R., Oakley H.A., McCracken V.J., Aminov R., Mackie R.I., Gaskins H.R. (2000). Molecular Ecological Analysis of the Succession and Diversity of Sulfate-Reducing Bacteria in the Mouse Gastrointestinal Tract. Appl. Environ. Microbiol..

[43] Yap C.X., Henders A.K., Alvares G.A., Wood D.L.A., Krause L., Tyson G.W., Restuadi R., Wallace L., McLaren T., Hansell N.K. (2021). Autism-Related Dietary Preferences Mediate Autism-Gut Microbiome Associations. Cell.

