# Supplementary material for: Desulfovibrio desulfuricans AY5 Isolated from a Patient with Autism Spectrum Disorder Binds Iron in Low-Soluble Greigite and Pyrite
Source: Microorganisms. 2021 Dec 10;9(12):2558. doi: 10.3390/microorganisms9122558 (PMC8705596; doi:10.3390/microorganisms9122558)
Supplement: Supplementary file 1 [file microorganisms-09-02558-s001.zip › microorganisms-1471128-supplementary.pdf]

Supplementary material to ***Desulfovibrio desulfuricans*** **AY5 isolated from a patient with autism spectrum disorders binds iron in low-soluble greigite and pyrite.**

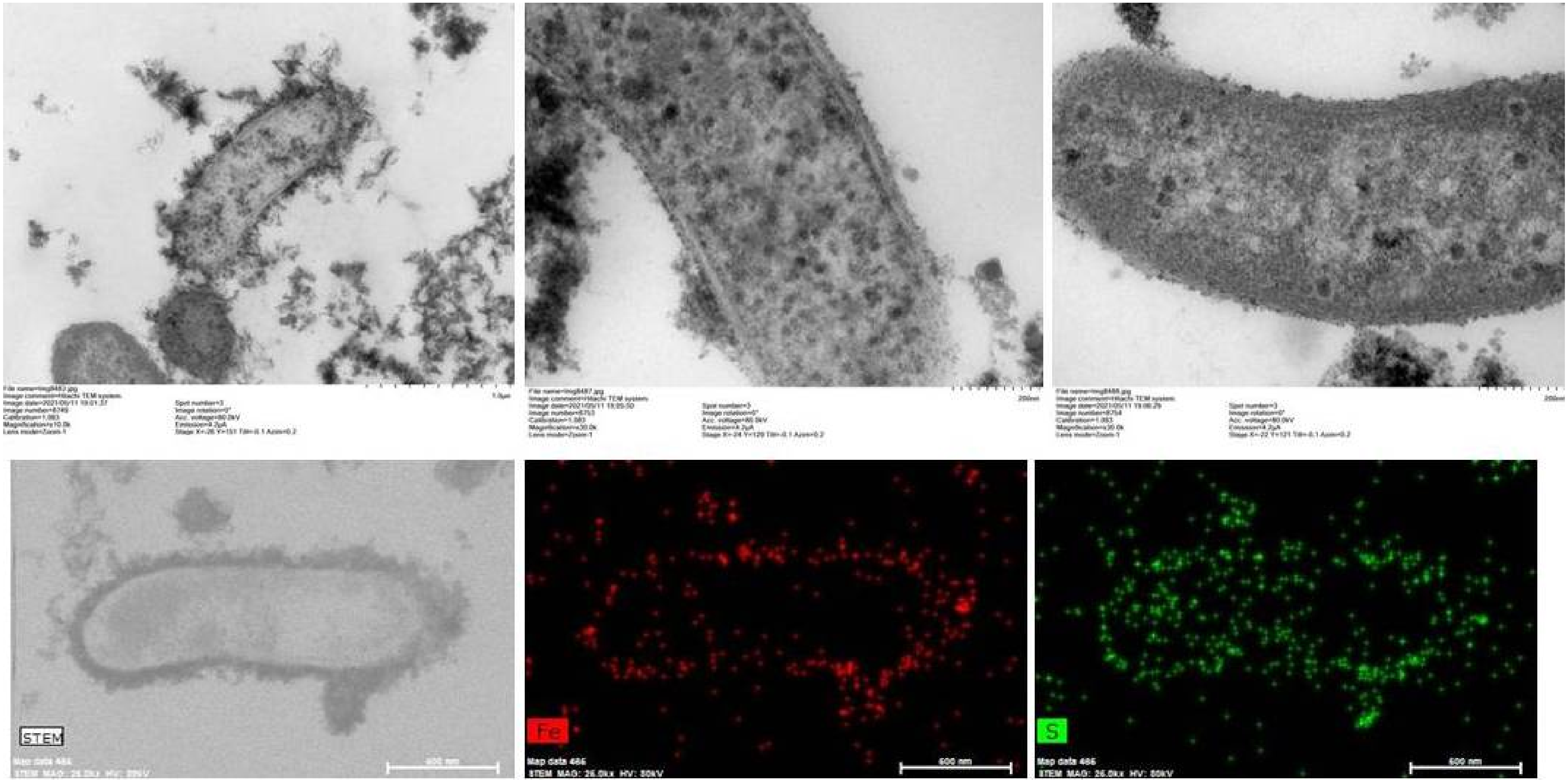

Figure S1. TEM micrograph of ultrathin cross section of strain AY5 cells on the second day of cultivation gown in the presence of 100 mg/L Fe showing electron-dense particles adhering on cell and inside cells and electron maps of Fe and S in the same image site with the same magnification .

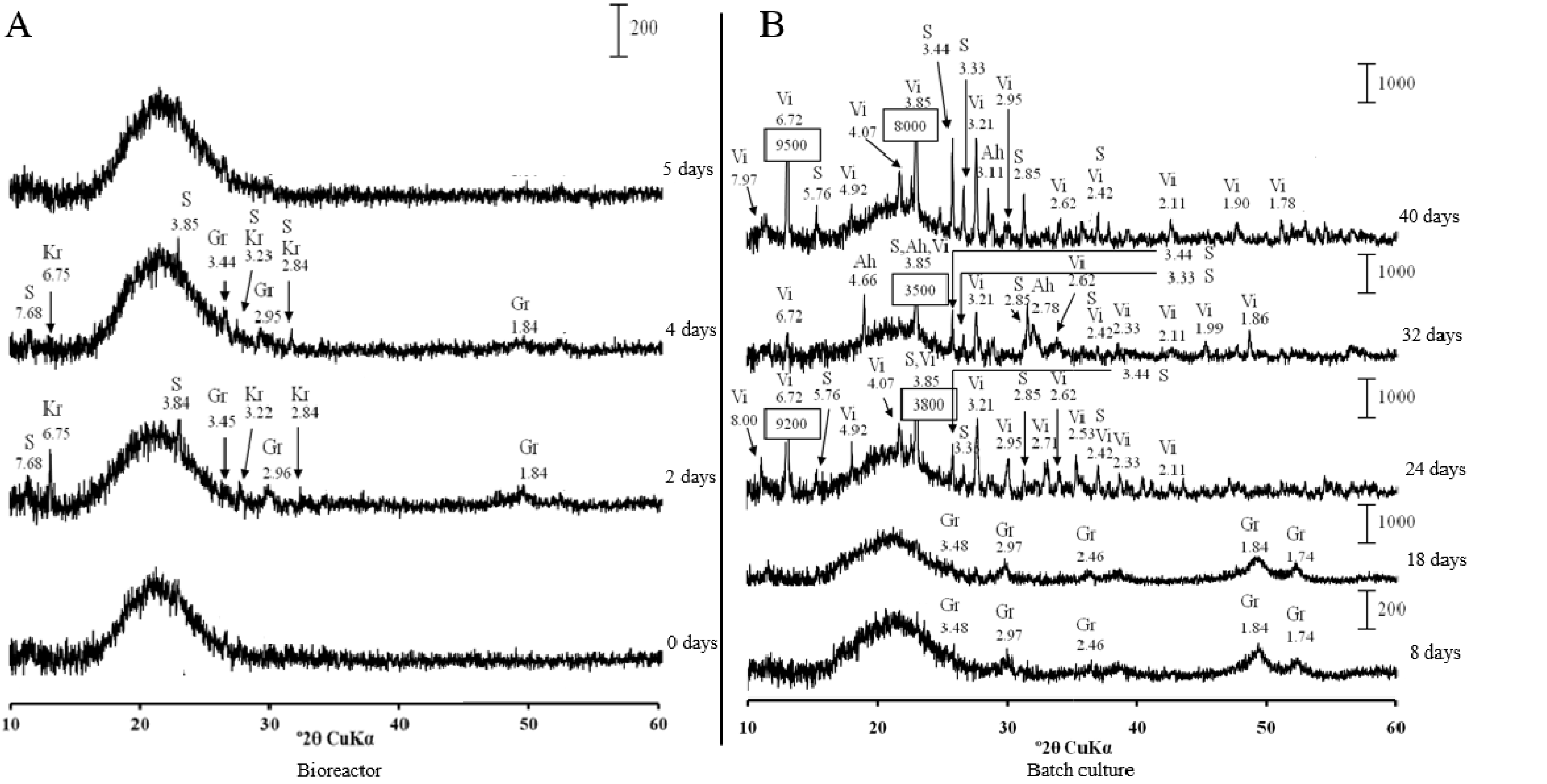

Figure S2. X-ray diffraction patterns of solids from bioreacttor (A) and batch cultures (B) of strainAY5 grown at pH6.5 after different incubation time. Letter cod greigite, Fe_3S_4 (PDF-16-0713), S = sulfur (PDF-06-0248), Kr = kornelite, $Fe_2^{+3}(SO_4)_3*7H_2O$ (PDF-44-1426), Vi = viviaannite, $Fe_3(PO_4)_2*8H_2O$ (PDF-30-0662).

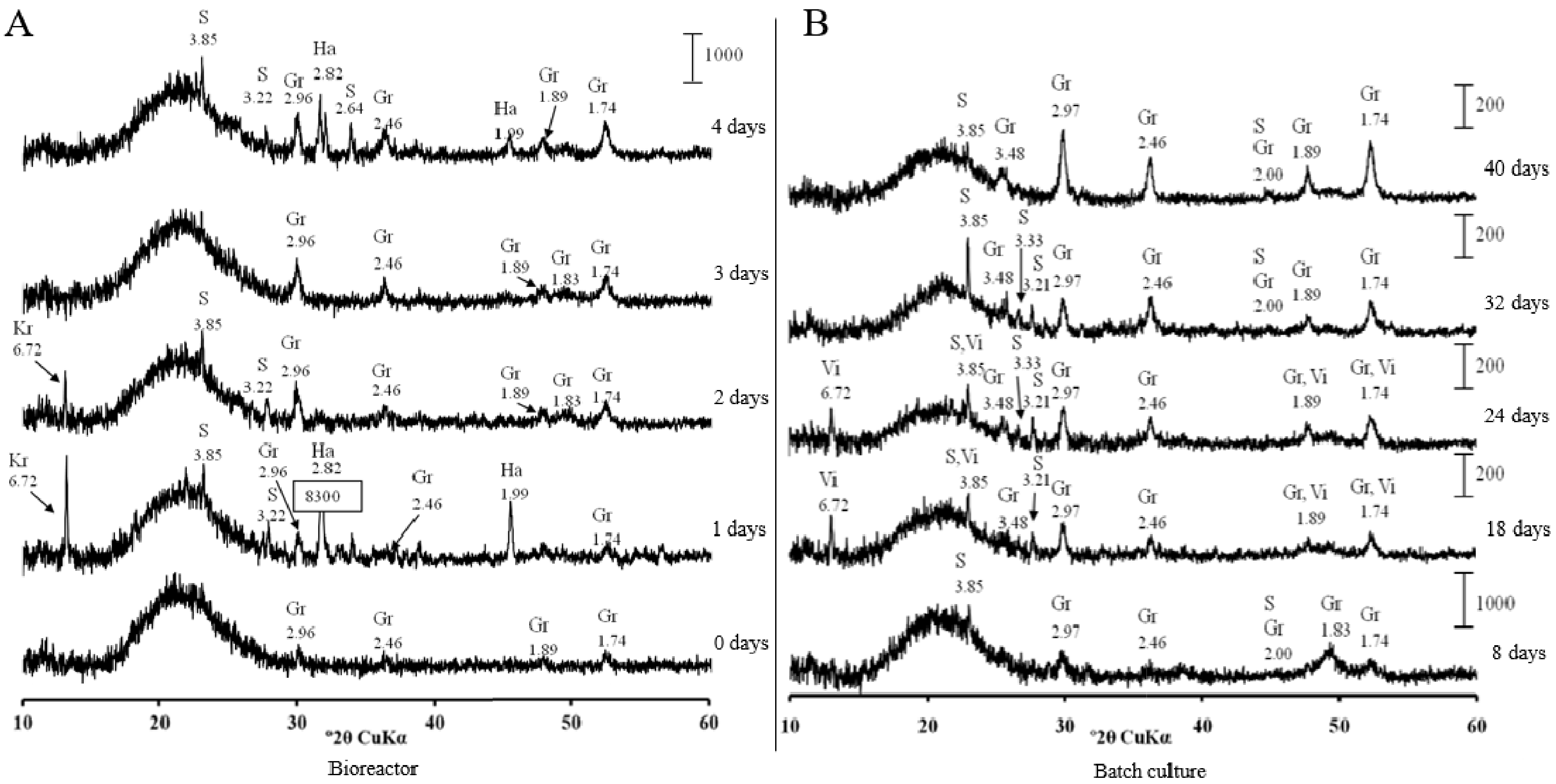

Figure S3. X-ray diffraction patterns of solids from bioreacttoor (A) and batch cultures (B) of strain AY5 grown at pH5.5 after different incubation time. Letter cod greigite, Fe_3S_4 (PDF-16-0713), S = sulfur (PDF-06-02448), Kr = kornelite, $Fe_2^{+3}(SO_4)_3*7H_2O$ (PDF-44-1426), Ha = halite, NaCl (PDF-05-0628), Vi = $vFe_3(PO_4)_2*8H_2O$ (PDF-30-0662).
